# Lymph node metastasis is not associated with survival in patients with clinical stage T4 esophageal squamous cell carcinoma undergoing definitive radiotherapy or chemoradiotherapy

**DOI:** 10.3389/fonc.2022.774816

**Published:** 2022-09-13

**Authors:** Liqiong Zhu, Zongxing Zhao, Ao Liu, Xin Wang, Xiaotao Geng, Yu Nie, Fen Zhao, Minghuan Li

**Affiliations:** ^1^ Department of Clinical Medicine, Shandong First Medical University and Shandong Academy of Medical Sciences, Jinan, China; ^2^ Department of Radiation Oncology, Shandong Cancer Hospital and Institute, Shandong First Medical University and Shandong Academy of Medical Sciences, Jinan, China; ^3^ Department of Radiation Oncology, Liaocheng People’s Hospital, Liaocheng, China; ^4^ School of Medicine, Shandong University, Jinan, China; ^5^ National Cancer Center/Cancer Hospital, Chinese Academy of Medical Sciences (CAMS) and Peking Union Medical College (PUMC), Beijing, China; ^6^ Department of Radiation Oncology, Weifang People’s Hospital, Weifang, China

**Keywords:** esophageal carcinoma, cT4 disease, tumor recurrence, prognosis, patient survival

## Abstract

**Background:**

Clinical T4 stage (cT4) esophageal tumors are difficult to be surgically resected, and definitive radiotherapy (RT) or chemoradiotherapy (dCRT) remains the main treatment. The study aims to analyze the association between the status of lymph node (LN) metastasis and survival outcomes in the cT4 stage esophageal squamous cell carcinoma (ESCC) patients that underwent treatment with dCRT or RT.

**Methods:**

This retrospective study analyzed the clinical data of 555 ESCC patients treated with dCRT or RT at the Shandong Cancer Hospital and the Liaocheng People’s Hospital from 2010 to 2017. Kaplan–Meier and Cox regression analyses was performed to determine the relationship between LN metastasis and survival outcomes of cT4 and non-cT4 ESCC patients. The chi-square test was used to evaluate the differences in the local and distal recurrence patterns in the ESCC patients belonging to various clinical T stages.

**Results:**

The 3-year survival rates for patients with non-cT4 ESCC and cT4 ESCC were 47.9% and 30.8%, respectively. The overall survival (OS) and progression-free survival (PFS) rates were strongly associated with the status of LN metastasis in the entire cohort (all *P* < 0.001) and the non-cT4 group (all *P* < 0.001) but not in the cT4 group. The local recurrence rates were 60.7% for the cT4 ESCC patients and 45.1% for the non-cT4 ESCC patients (*P* < 0.001). Multivariate analysis showed that clinical N stage (*P* = 0.002), LN size (*P* = 0.007), and abdominal LN involvement (*P* = 0.011) were independent predictors of favorable OS in the non-cT4 group. However, clinical N stage (*P* = 0.824), LN size (*P* = 0.383), and abdominal LN involvement (*P* = 0.337) did not show any significant correlation with OS in the cT4 ESCC patients.

**Conclusions:**

Our data demonstrated that the status of LN metastasis did not correlate with OS in the cT4 ESCC patients that received dCRT or RT. Furthermore, the prevalence of local recurrence was higher in the cT4 ESCC patients.

## Introduction

Esophageal carcinoma (EC) is the sixth leading cause of cancer-related mortality worldwide (1). Esophageal squamous cell carcinoma (ESCC) is the main EC type in Asia and South America, whereas esophageal adenocarcinoma is the most frequent type of EC in Europe and the USA (2). The low survival rate of ESCC patients is primarily attributed to diagnosis in the advanced stages (3). The clinical T4 (cT4) EC tumors are characterized by tumor invasion into the adjacent anatomical structures. Despite significant advances in the surgical techniques, cT4 ESCC is considered inoperable. Currently, definitive chemoradiotherapy (dCRT) or radiotherapy (RT) is the standard therapy for ESCC patients who refuse surgery or are ineligible for surgical resection (4, 5). It is a clinical challenge to determine the clinical target volume (CTV) of elective nodal irradiation (ENI) or involved-field radiotherapy (IFRT) Furthermore, the optimal RT strategy for EC patients at different clinical T stages is unclear.

The number of metastatic lymph node (LN) is associated with survival of ESCC patients that have undergone surgery (6, 7). However, the effect of LN metastasis status on the survival for the non-surgical ESCC patients remains unclear, especially those in the cT4 stage. In order to provide clinical information to individualized RT strategies for different cT stages, we investigated the relationship between the status of LN metastasis and the survival outcomes in the cT4 and non-cT4 ESCC patients.

## Methods

### Patients

The clinical data of 555 patients with ESCC without distant metastasis who were treated with dCRT or RT at the Shandong Cancer Hospital and the Liaocheng People’s Hospital between April 2010 and December 2017 was analyzed retrospectively. The pre-treatment staging was based on data from the physical examinations, barium swallow test, tissue biopsy, and imaging data from endoscopic ultrasonography, contrast-enhanced computed tomography (CT), and/or 18F-fluorodeoxyglucose imaging (18F-FDG). Tumor staging was performed by three experienced radiologists based on the guidelines from the American Joint Committee on Cancer (AJCC) Cancer Staging Manual (Eighth edition) (8). This study included ESCC patients with the cT2–4 stages that were classified into the following three groups: (1) Total (cT4+non-cT4) group; (2) cT4 group, and (3) non-cT4 group. Patients with metastatic LNs > 2 cm showed higher rates of tumor recurrence and poor treatment response (9). The extent of LN involvement was classified into three groups based on the number of anatomical positions (cervical, thoracic, and abdomen) involved. Then, the relationship between patient survival and the status of LN metastasis was analyzed. The status of LN metastasis was based on multiple characteristics, namely, (1) the number of metastatic LNs: cN0, cN1, cN2 and cN3; (2) the extent of LN metastasis: cN0, involvement of one anatomical region, involvement of two anatomical regions, and involvement of three anatomical regions; (3) LN size: cN0, ≤2 cm, and >2 cm; (4) abdominal LN involvement: cN0, with or without abdominal LN involvement. This study was approved by the Medical Ethics Committees of the Shandong Cancer Hospital and the Liaocheng People’s Hospital.

### Criteria for LN metastasis and cT4 stage

In this study, the cT4a stage was defined as tumor invasion into the pleura, pericardium, azygos vein, diaphragm, or the peritoneum. The cT4b stage was defined as tumor invasion into additional surrounding structures such as the aorta, the vertebral body, and the trachea (10). Patients with cT4 stage demonstrated loss of fat plane between the primary tumor and the adjacent mediastinal structures (11). Tracheobronchial invasion was defined as a tumor protrusion into the lumen of the trachea or the bronchus on the CT scans. Aortic invasion was defined as > 90 degree contact between the aorta and the tumor or loss of fat plane in the triangular space between the esophagus, spine, and the aorta on the CT scans (12).

Regional LNs were defined as those in the periesophageal tissue from the upper esophageal sphincter to the adventitia of the celiac artery as previously described (10). LN metastasis was confirmed as positive if the short axis diameter of the LNs was >10 mm or if the short axis diameter of the paraesophageal, tracheoesophageal sulcus, pericardial angle, or the abdominal LNs was > 5 mm in the CT or magnetic resonance imaging (MRI) scans. LNs were considered as metastatic if they demonstrated a round shape, hypoechoic pattern, and visible borders in the endoscopic ultrasonography, or demonstrated high maximum standardized FDG uptake. The diagnostic accuracy of CT in the cT4 esophageal cancer patients was 80%. In this study, CT examination was used to diagnose all the patients with cT4 ESCC.

### Treatment details

The gross tumor volume (GTV) was delineated on the 3-dimensional (3D) planning system by the supervising radiation oncologist using data from the CT/MRI fusion scans, diagnostic CT scans, and endoscopic ultrasonography scans. The GTV included all the visible macroscopic esophageal lesions and the clinically positive LNs. The CTV included 3–5 cm cephalic and caudal margins of the primary tumor, 0.8–1.0 cm radial margins, and the regional high-risk LNs. The CTV for IFI included the clinically positive lymph nodes with metastasis and the CTV for ENI included regional high-risk LNs. Most patients were treated with ENI in our study. The organs at risk, including the heart, spinal cord, and the lungs, were outlined. All the patients received a total dose of 50.4–66 Gy in 28–33 fractions (1.8/2.0-Gy fractions once daily, 5 days a week). All the patients received 3D-conformal or intensity-modulated RT. In this study, 309 patients received concurrent platinum-based chemotherapy and 246 patients received definitive RT alone due to advanced age, complications, or refusal to receive chemotherapy. All the patients underwent dCRT received at least 2 cycles of chemotherapy (a combination of platinum and 5-fluorouracil or platinum and taxanes or other commonly used protocols).

### Follow up

The patients were examined 1-month after treatment completion, every 3 months for the first 2 years, and every 6 months until loss of follow-up or death. Each follow-up assessment included a physical examination, blood test, esophageal endoscopy, enhanced CT scans, and the barium swallow test. The patients who missed their follow-up schedule were sent reminders by phone. Patients with suspected recurrence were subjected to histology or cytology testing. Local recurrence was defined as recurring tumor at the primary tumor site or the regional LNs. Recurrence at any other site was defined as distant recurrence. Overall survival (OS) and progression-free survival (PFS) were calculated from the day of pathologic diagnosis until an event or censorship.

### Statistical analysis

The statistical analysis in this study was performed using the SPSS version 24.0 software (SPSS, Chicago, IL, USA). Survival analysis was performed using the Kaplan–Meier method. The chi-square test was used to compare the differences in local recurrence and distant recurrence between the cT4 and non-cT4 patients with ESCC. Log-rank tests and Cox proportional risk regression models were used to assess the relationship between patient survival and the clinical factors. A two-sided p-value <0.05 was considered statistically significant.

## Results

### Patient characteristics

This retrospective study analyzed the clinical data of 555 ESCC patients that were treated between April 2010 and December 2017 at the Shandong Cancer Hospital (n=406) and the Liaocheng People’s Hospital (n=149). Among these, 107 (19.3%) patients were diagnosed with the cT4 stage disease and 448 (80.7%) patients were diagnosed with the non-cT4 stage disease. The median follow-up time was 41.5 months. The median age at diagnosis was 66 years (range: 38–90 years) and included 421 (75.9%) males and 134 (24.1%) females. Furthermore, 309 (55.7%) patients received dCRT and 246 (44.3%) patients received definitive RT alone. LN metastasis was diagnosed in 394 (71.0%) patients. Moreover, 161 (29.0%), 211 (38.0%), 152 (27.4%), and 31 (5.6%) patients were classified as cN0, cN1, cN2, and cN3, respectively. The detailed characteristics of the included patients are listed in **Table 1**.

**Table 1:** Total group (cT2-4) patients’ characteristics of prognostic factors.

Variables	Total Group (T2/T3/T4)	non-cT4 Group (T2/T3)	cT4 Group	P Value
**All**	555	448 (80.7%)	107 (19.3%)	
**Age (years)**				**0.020**
≤60 years	143 (25.8%)	106 (23.7%)	37 (34.5%)	
> 60 years	412 (74.2%)	342 (76.3%)	70 (65.5%)	
**Sex, n (%)**				0.142
Female	134 (24.1%)	114 (25.4%)	20 (18.6%)	
Male	421 (75.9%)	334 (74.6%)	87 (81.4%)	
**Smoking, n (%)**				0.879
Never	263 (47.4%)	213 (47.5%)	50 (46.7%)	
Ever	292 (52.6%)	235 (52.5%)	57 (53.3%)	
**Drinking, n (%)**				**0.031**
Never	311 (56.0%)	261 (58.3%)	50 (46.7%)	
Ever	244 (44.0%)	187 (41.7%)	57 (53.2%)	
**Tumor location, n (%)**				0.762
Cervical Upper	243 (43.8%)	199 (44.4%)	44 (41.1%)	
Middle	190 (34.2%)	153 (34.2%)	37 (34.6%)	
Lower	122 (22.0%)	96 (21.4%)	26 (24.3%)	
**Treatment regimen,** **n (%)**				0.757
RT alone	246 (44.3%)	200 (44.6%)	46 (42.9%)	
CRT	309 (55.7%)	248 (55.3%)	61 (57.1%)	
**RT dose, n (%)**				**0.044**
≤60 Gy	397 (71.5%)	312 (69.6%)	85 (79.5%)	
> 60 Gy	158 (28.5%)	136 (30.3%)	22 (20.5%)	
**Clinical cN**				0.380
cN0	161 (29.0%)	135 (30.1%)	26 (24.3%)	
cN1	211 (38.0%)	168 (37.5%)	43 (40.2%)	
cN2	152 (27.4%)	123 (27.5%)	29 (27.1%)	
cN3	31 (5.6%)	22 (4.9%)	9 (8.4%)	
**Extent of LNs**				0.667
0	161 (29.0%)	135 (30.1%)	26 (24.3%)	
1 region	258 (46.5%)	202 (45.1%)	54 (50.4%)	
2 regions	112 (20.2%)	90 (20.1%)	22 (20.6%)	
3 regions	24 (4.3%)	21 (4.7%)	5 (4.7%)	
**Size of LNs**				0.104
0	161 (29.0%)	135 (30.1%)	26 (24.3%)	
≤ 2 cm	329 (59.3%)	268 (59.8%)	63 (58.9%)	
>2 cm	65 (11.7%)	45 (10.1%)	18 (16.8%)	
**Abdominal region-involved**				0.490
N0	161 (29.0%)	135 (30.2%)	26 (24.3%)	
Without	322 (59.0%)	255 (56.9%)	66 (61.7%)	
With	72 (13.0%)	58 (12.9%)	15 (14.0%)	

LNs, lymph nodes; RT, chemoradiotherapy; CRT, chemoradiotherapy; cT4, clinical T4; Bold values indicates a statistically difference in statistical analysis.

### Recurrence patterns in cT4 ESCC patients compared to the non-cT4 ESCC patients

The follow-up showed that the prevalence of local recurrence was 48.1% (267 cases), the prevalence of distant recurrence was 22.8% (127 cases) in all the included ESCC patients, and 7.6% (42 cases) both in. The relationship between the clinical T stages and the tumor recurrence patterns is shown in **Table 2**. Local recurrence was significantly higher in patients with cT4 disease compared to those with non-cT4 ESCC (60.7% vs. 45.1%; *P <*0.001). However, distant recurrence rates were statistically insignificant between cT4 ESCC and non-cT4 ESCC patients (26.2% vs. 22.1%; *P* = 0.368).

**Table 2:** Correlation between cT stage and patterns of failure.

Patterns of failure	cT4	non-cT4	P value
**LR**			**<0.001**
yes	65 (60.7%)	202 (45.1%)	
no	42 (39.3%)	246 (54.9%)	
**M**			0.368
yes	28 (26.2%)	99 (22.1%)	
no	79 (73.8%)	349 (77.9%)	

LR, local recurrence; M, metastases; cT4, clinical T4; Bold values indicates a statistically difference in statistical analysis.

### Survival

The 1-, 3-, and 5-year OS rates for all the included patients in this study were 80.9%, 44.8%, and 33.3%, respectively. The median survival time was 30.2 months (range: 1.7-82.2 months). The 3-year OS rates in all patients with cN0, cN1, cN2, and cN3 disease were 56.9%, 46.2%, 37.8%, and 0%, respectively. The median OS rates in all patients with cN0, cN1, cN2, and cN3 disease were 46.0, 31.0, 26.0, and 11.0 months, respectively.

### Effect of lymph node metastasis status on the survival outcomes

**Figure 1:**
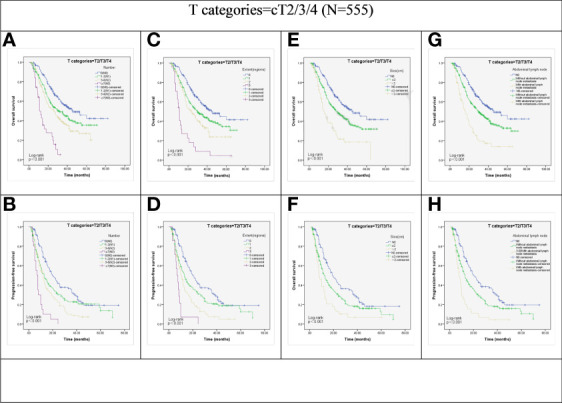
Kaplan-Meier representations of OS and PFS with respect to the N stage **(A, B)**, lymph node extent **(C, D)**, lymph node size **(E, F)**, and abdominal lymph node **(G, H)** in cT2/3/4 patients (N=555). P values were all less than 0.001.

Kaplan–Meier survival analysis demonstrated that factors such as N stage (cN0, cN1, cN2 and cN3), extent of LN metastasis (cN0, 1 region, 2 regions, and 3 regions), size of the LNs (cN0, 2cm, and ≥ 2cm), and abdominal LN metastasis (cN0, with or without abdominal involvement) showed significant correlation with OS and PFS in the total (cT4+non-cT4) group (all *P* < 0.001; **Figure 1**).

**Figure 2:**
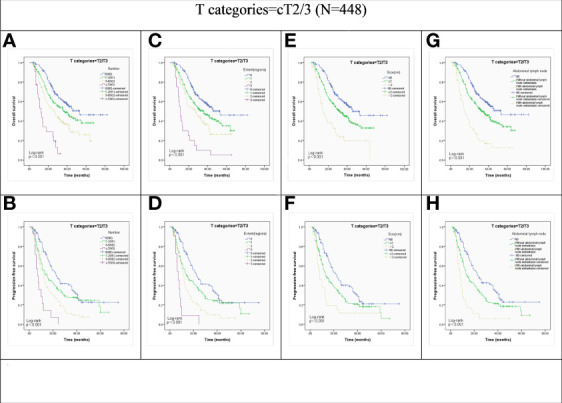
Kaplan-Meier representations of OS and PFS with respect to the N stage **(A, B)**, lymph node extent **(C, D)**, lymph node size **(E, F)**, and abdominal lymph node **(G, H)** in cT2/3 patients (N=448). P values were all less than 0.001.

We then investigated the effect of the LN metastasis status on the survival outcomes in different clinical T stages. The advanced N stage, larger LNs, extent of LN metastasis, and presence of abdominal LN metastasis showed significant correlation with poor OS and PFS in the non-cT4 group (all *P* < 0.001; **Figure 2**).

**Figure 3:**
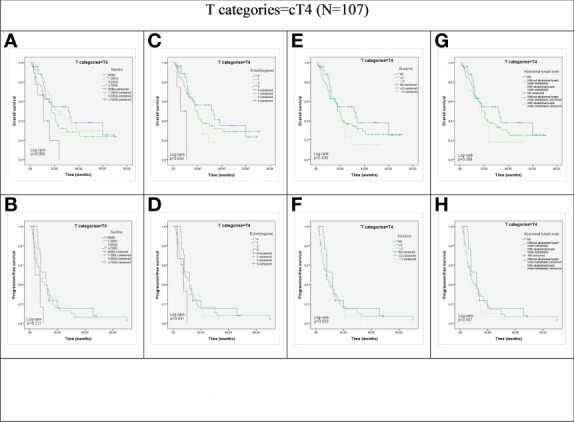
Kaplan-Meier representations of OS and PFS with respect to the N stage (**(A)** p=0.059; **(B)** p=0.121), lymph node extent (**C**, p=0.034; **(D)** p=0.431), lymph node size (**(E)**, p=0.430; **(F)**, p=0.650), and abdominal lymph node (**(G)**, p=0.399; **(H)**, p=0.547) in cT4 patients (N=107).

However, in patients with cT4 disease, OS and PFS did not show significant association with the N stage (*P* = 0.059 and *P* = 0.121; **Figures 3A, B**), LN size (*P* = 0.430 and *P* = 0.650; **Figures 3E, F**), and the abdominal LN involvement (*P* = 0.399 and *P* = 0.547; **Figures 3G, H**). Although the extent of LN metastasis showed a significant association with the OS of cT4 group patients (*P* =0.034; **Figure 3C**), the Kaplan-Meier curve analysis showed the survival curves of patients in cN0, 1, 2, and 3 anatomic regions were crossed. Furthermore, there was no significant association between the extent of LN metastasis and progression-free survival (PFS) in the cT4 group (*P* = 0.431; **Figure 3D**).

### Prognostic factors

Univariate analysis was performed to identify clinical factors associated with prognostic prediction in the total (cT4+non-cT4) group, the non-cT4 group and the cT4 groups. Then, factors with *P*-values < 0.2 were included in the multivariate analysis and the results are shown in **Table 3**. Of note, the lymph node metastasis status as a prognostic factor differed among the total group, non-cT4 and cT4 ESCC group. The clinical N stage (hazard ratio [HR], 1.534; 95% CI, 1.189–1.980; *P* = 0.001), LN ≥2 cm (HR, 1.502; 95% CI, 1.060–2.129; *P* = 0.022), and abdominal LN metastasis (HR, 1.462; 95% CI, 1.026–2.085; *P* = 0.036) were independent predictors of favorable OS for the total group. Furthermore, clinical N stage (HR, 1.599; 95% CI, 1.195–2.140; *P* = 0.002), LN ≥2 cm (HR, 1.737; 95% CI, 1.167–2.587; *P* = 0.007), and abdominal LN involvement (HR, 1.681; 95% CI, 1.126–2.512; *P* = 0.011) were independent predictors of favorable OS in patients with non-cT4 disease. However, none of these node-related factors were independent predictors of OS in patients with cT4 ESCC.

**Table 3:** Cox regression of OS for total group (cT2/3/4), non-T4 group(cT2/3) and cT4 group ESCC patients.

Variables	Total Group (cT2/T3/T4)				non-T4 Group (cT2/T3)				cT4 Group			
	Univariate analysis		Multivariate analysis		Univariate analysis		Multivariate analysis		Univariate analysis		Multivariate analysis	
	HR (95% CI)	P value	HR (95% CI)	P value	HR (95% CI)	P value	HR (95% CI)	P value	HR (95% CI)	P value	HR (95% CI)	P value
**Sex**												
Female	1				1				1			
Male	1.342 (1.015-1.774)	**0.039**		NS	1.272 (0.937-1.727)	**0.123**		NS	1.478 (0.731-2.989)	0.277		
**Age**												
≤60 years	1				1				1			
> 60 years	1.073 (0.828-1.390)	0.594			1.159 (0.856-1.571)	0.340			0.999 (0.602-1.658)	0.996		
**Smoking**												
Never	1				1				1		1	
Ever	1.348 (1.073-1.694)	**0.010**		NS	1.239 (0.958-1.603)	**0.102**		**NS**	1.829 (1.107-3.022)	**0.018**	1.829 (1.107-3.022)	**0.018**
**Drinking**												
Never	1		1		1		1		1			
Ever	1.440 (1.149-1.803)	**0.002**	1.424 (1.134-1.789)	**0.002**	1.315 (1.019-1.698)	**0.035**	1.334(1.031-1.726)	**0.028**	1.736 (1.049-2.872)	**0.032**		**NS**
**Tumor location**		**<0.001**		**0.001**		**<0.001**		**0.007**		**0.105**		**NS**
Cervical/Upper	1		1		1		1		1			
Middle	1.483 (1.146-1.945)	**0.003**	1.391 (1.063-1.820)	**0.016**	1.514 (1.123-2.040)	**0.006**	1.362 (1.003-1.848)	**0.048**	1.347 (0.759-2.391)	0.308		
Lower	2.238 (1.681-2.980)	**<0.001**	1.818 (1.323-2.499)	**<0.001**	2.292 (1.658-3.169)	**<0.001**	1.774 (1.240-2.540)	**0.002**	1.950 (1.053-3.611)	**0.034**		
**RT dose**												
≤60 Gy	1				1				1			
>60 Gy	0.723 (0.557-0.939)	**0.015**		NS	0.770 (0.578-1.025)	**0.073**		NS	0.624 (0.318-1.225)	**0.171**		**NS**
**Treatment regimen**												
RT alone	1		1		1		1		1			
CRT	0.854(0.680-1.071)	**0.171**	0.744 (0.589-0.940)	**0.013**	0.806 (0.624-1.041)	**0.099**	0.689(0.528-0.898)	**0.006**	0.989 (0.599-1.634)	0.967		
**Clinical N stage**												
cN0-1	1		1		1		1		1			
cN2-3	1.733(1.324-2.267)	**<0.001**	1.534(1.189-1.980)	**0.001**	1.911(1.474-2.276)	**<0.001**	1.599(1.195-2.140)	**0.002**	1.060(0.637-1.762)	0.824		
**Extent of LNs**												
0-1 region	1				1				1			
2-3 regions	1.791(1.403-2.302)	**<0.001**		NS	1.871(1.419-2.468)	**<0.001**		NS	1.506(0.859-2.642)	**0.153**		**NS**
**Size of LNs**												
≤ 2 cm	1		1		1		1		1			
>2 cm	2.160(1.566-2.979)	**<0.001**	1.502(1.060-2.129)	**0.022**	2.415(1.668-3.495)	**<0.001**	1.737(1.167-2.587)	**0.007**	1.336(0.696-2.564)	0.383		
**Abdominal region-involved**												
NO	1		1		1		1		1			
YES	2.411(1.784-3.258)	**<0.001**	1.462(1.026-2.085)	**0.036**	2.753(1.967-3.853)	**<0.001**	1.681(1.126-2.512)	**0.011**	1.392(0.709-2.737)	0.337		

LNs, lymph nodes; CI, confidence interval; CRT, chemoradiotherapy; RT, radiotherapy; HR, hazard ratio; NS, non-significant. Bold values indicate a statistically difference in statistical analysis.

## Discussion

This study investigated the association between LN metastasis and survival outcomes in ESCC patients who underwent RT and the prognostic differences between ESCC patients belonging to the cT4 and non-cT4 stages. The study cohort of ESCC patients was categorized into sub-groups based on multiple criteria such as the number of metastatic LNs according to the AJCC recommendations, the size of LNs, and others. The results of this study highlighted significant differences in the survival outcomes of ESCC patients based on the metastatic LN status.

Tumor staging is essential for determining the optimal treatment strategy for the EC patients. Previous studies reported the association between the number of metastatic LNs and the survival outcomes of ESCC patients that underwent esophagectomy (13, 14). This study investigated the correlation between the number of metastatic LNs and the survival outcomes of ESCC patients receiving RT according to the AJCC guidelines for EC. The results showed significant differences in the survival outcomes between the cT4 and the non-cT4 ESCC patients. Our data demonstrated that the advanced N stage non-cT4 patients were associated with worse OS and PFS. However, we did not observe the association between N stages and the survival outcomes in the cT4 ESCC patients. Furthermore, the prognostic value of N staging based on the number of metastatic LNs is a matter of debate for EC patients that underwent surgery (15–17). Therefore, in the clinic, other LN staging strategies have been used for the ESCC patients.

The size of LNs is one of the factors used for N staging in the non-surgically treated patients with head and neck tumors. It has been demonstrated that the size of LNs correlates with treatment outcomes and prognostic prediction in EC patients treated surgically (18–20). Furthermore, in our previous study regarding ESCC patients treated with RT, the objective response rates (ORRs) of patients with metastatic LNs > 2cm were significantly worse compared to those with metastatic LNs ≤ 2 cm (*P* = 0.038) (9). In ESCC, the size of LNs shows significant prognostic value and positive correlation with the extra-nodal spread (21, 22). Therefore, we evaluated the relationship between LN metastasis status and the survival outcomes using the size of LNs in patients with ESCC at different cT stages as a parameter. We observed significant differences in the OS and PFS rates of patients belonging to the N0, LN ≤ 2cm, LN > 2cm categories in the non-cT4 ESCC group. However, it was not observed that the correlation between the size of LNs and the survival outcomes had significant differences in the cT4 ESCC group.

The frequency of cross-regional LN metastasis is high in ESCC because of the abundant lymphatic channels in the lamina propria and submucosa of the esophagus. Several studies have investigated the association between the anatomical regions of LN metastasis and the survival outcomes of patients with ESCC, and recommend the extent of lymph node metastases as the basis for N staging (15, 23, 24). Our results demonstrated that the OS and PFS rates varied significantly in regard to the various extent (1,2,3 anatomical regions involved) of LN metastasis in the non-cT4 group. However, the various extent of LN metastasis was not associated with survival prediction in the ESCC patients of the cT4 group.

Abdominal LN metastasis was considered as distant metastasis in the 6th edition of the AJCC/UICC TNM staging guidelines and as regional metastasis in the 7th and 8th editions of AJCC/UICC TNM staging guidelines. Rutegard et al. (25) retrospectively analyzed 446 patients with stage III or IV EC that underwent surgical resection and demonstrated that the disease-specific mortality and OS rates of patients with celiac LN metastasis were comparable to those of patients with metastasis to the distant organs. Therefore, in this study, we stratified the ESCC patients into groups according to the presence of metastatic lesions in the abdominal LNs, which included LNs in the paracardial, left gastric artery, common hepatic artery, splenic artery, and the celiac trunk. Our results showed significant differences in the OS and PFS rates between non-cT4 patients with or without abdominal LN metastasis (all *P* < 0.001), but the differences in OS (*P* =0.399) and PFS (*P* =0.547) rates were not statistically significant between cT4 patients with or without abdominal LN metastasis.

Local recurrence was predominant among the ESCC patients in this study. Moreover, the prevalence of local recurrence was significantly higher in patients with cT4 ESCC compared to the patients with non-cT4 ESCC. Welsh et al. (26) reported that the T stage of patients with EC undergoing dCRT was associated with local control, and the local control rate in patients with T3 and T4 tumors was significantly lower than that of patients with T1 and T2 tumors (25% vs. 71%). Another study also confirmed more frequent local recurrence at the primary tumor site compared to the LNs in the EC patients receiving dCRT (27). Our data was consistent with results of the previously reported findings (26–28) and showed that local recurrence was more frequent than distant recurrence (outfield of the planning target volume recurrence) in EC patients treated with RT, and advanced T-stages were associated with poor local tumor control. Our preliminary data showed that the median PFS for primary progression in the cT4 ESCC patients was 7.63 months (range: 1.5-70 months) and 8.5 months (range: 2–32.87 months) for patients with distant metastasis. This suggested that local recurrence often preceded distant recurrence among the cT4 ESCC patients treated with RT. The effect of T stage may mask the impact of LN metastasis on the survival outcomes of the cT4 ESCC patients. Therefore, local control should be prioritized over irradiating distant LNs in such patients. Hence, local salvage treatments such as re-RT or salvage esophagectomy may be beneficial for ESCC patients with advanced T staging.

The CTV coverage for elective nodal irradiation (ENI) and involved-field radiotherapy (IFRT) is not clear in the advanced T-stage ESCC patients. Several studies have reported that ENI prevented regional LN recurrence but did not improve OS and local control of ESCC patients (29–31). Moreover, regional LN failure was not common in ESCC patients that received ENI or IFRT (32, 33). Our findings showed that the pre-treatment LN metastasis status did not correlate with the survival outcomes of cT4 ESCC patients. Therefore, we postulate that prophylactic LN irradiation may not significantly improve survival. Furthermore, IFRT was associated with reduced lung, esophagus and hematological toxicity, thereby enabling a higher number of EC patients to tolerate RT and chemotherapy (29). Because of the advanced disease stage and high local recurrence rates, involved-field irradiation (IFI) may be more beneficial for the cT4 ESCC patients.

This study has several limitations. First, this was a retrospective study that included patients with significantly different treatment plans including radiation doses and chemotherapy regimens. Second, LN metastasis in the study cohort was assessed by the non-invasive pre-processing staging methods such as endoscopic ultrasonography (EUS), computed tomography (CT), and fluorodeoxyglucose-positron emission tomography (FDG-PET) rather than histopathology methods. Third, the competitive risk model may be better at estimating the impacts of local and distal recurrence on LN metastasis and survival.

## Conclusions

The status of LN metastasis characteristics such as the number of metastatic LNs, size of the metastatic LNs, and abdominal LN metastasis was associated with the survival prediction of patients with non-cT4 ESCC who have received radiotherapy. However, LN metastasis status was not associated with the survival outcomes of patients with cT4 ESCC. Our results suggested that the treatment strategy for cT4 ESCC patients may be different from the treatment strategy for the non-cT4 ESCC patients and may require strengthening the local control of the primary lesions for cT4 ESCC patients. Future prospective and randomized clinical trials are required to validate the feasibility and efficacy of high radiation doses and IFRT in patients with non-cT4 ESCC.

## Data availability statement

The original contributions presented in the study are included in the article/supplementary material. Further inquiries can be directed to the corresponding authors.

## Author contributions

LZ, ZZ, FZ and ML analyzed the data and drafted the manuscript. AL, XW, XG, and YN participated in data collection. All authors read and approved the final manuscript.

## Funding

This study was supported by the Natural Science Foundation of China (Grant No NSFC 82172677).

## Acknowledgments

The authors would such as to express their great thanks to the Natural Science Foundation of China.

## Conflict of interest

The authors declare that the research was conducted in the absence of any commercial or financial relationships that could be construed as a potential conflict of interest.

## Publisher’s note

All claims expressed in this article are solely those of the authors and do not necessarily represent those of their affiliated organizations, or those of the publisher, the editors and the reviewers. Any product that may be evaluated in this article, or claim that may be made by its manufacturer, is not guaranteed or endorsed by the publisher.
